# Psychosocial correlates of mental health of university students in Hong Kong under COVID-19

**DOI:** 10.3389/fpsyg.2023.1294026

**Published:** 2023-11-22

**Authors:** Daniel T. L. Shek, Wenyu Chai, Diya Dou, Xiang Li, Cathy H. M. Chan

**Affiliations:** Department of Applied Social Sciences, The Hong Kong Polytechnic University, Kowloon, Hong Kong SAR, China

**Keywords:** psychological morbidity, COVID-19 stress, psychosocial correlates, university students, COVID-19 pandemic

## Abstract

Although the COVID-19 pandemic has caused many problems for university students, there are several research gaps in the study of psychological well-being of Hong Kong university students. First, few studies have examined different ecological correlates of mental health in a single study. Second, few studies have used both psychological morbidity and positive well-being as indicators of mental health. Third, we know little about the relationships between university students’ perceived need satisfaction, difficulties, service utilization, and their mental health. Hence, we conducted a study (*N* = 1,020 university students) in the later stage of the COVID-19 pandemic in Hong Kong. For mental health, we included measures of negative mental health (psychological morbidity) and positive mental health. We addressed several research questions in this study: (1) what is the mental health status of Hong Kong university students? (2) what is the relationship between COVID-19 stress and student mental health? (3) what are the intrapersonal correlates of student mental health? (4) are interpersonal factors related to student mental health? (5) are need satisfaction, difficulties encountered, and service utilization related to students’ mental health? (6) are there gender differences in the effects of correlates in different ecological systems? Analyses using structural equation modeling showed several observations. First, the prevalence of mental health symptoms among university students was alarming. Second, COVID-19 related socio-economic stress positively predicted psychological morbidity but negatively predicted well-being. Third, beliefs about adversity, resilience, and emotional competence predicted mental health. Fourth, family functioning was related to psychological morbidity negatively but connected with well-being positively. Fifth, while need satisfaction predicted psychological morbidity negatively, difficulties encountered showed the opposite direction. Besides, the perceived usefulness of university services positively predicted mental health. Finally, there were no gender differences in the effects of different predictors. The present findings enable public health researchers to formulate theoretical models on different ecological determinants of university students’ mental health under the pandemic. For public health practitioners, the study highlights the importance of reducing COVID-19 associated stress, strengthening internal and external developmental assets, and meeting the psychosocial needs of university students as strategies to promote their mental health under the pandemic.

## 1 Introduction

University students’ mental health has drawn increasing attention in research in recent years (Lipson et al., 2019). Many university students face heavy academic and financial pressure, which often results in poorer academic and adjustment problems (Seki et al., 2019; Paleari et al., 2021). With the onset of COVID-19, the pandemic has adversely affected many people including university students, such as triggering the “COVID-19 stress syndrome” (Taylor et al., 2020) and “COVID-19 anxiety syndrome” as a dysfunctional cognitive-behavioral coping strategy to respond to the threat of the pandemic (Mansueto et al., 2022; Alhakami et al., 2023), leading to prolonged mental health problems such as depressive symptoms, post-traumatic stress disorder (PTSD), problematic internet use and suicidal ideation (Kennedy et al., 2021; Magson et al., 2021). Besides, the pandemic has also led to some somatic symptoms such as fatigue and emotional distress including negative mood and loneliness (Walton et al., 2020; Mansueto et al., 2021). Compared with the general population, university students might suffer more from the impact of the pandemic due to their being in a stage transiting from late adolescence to early adulthood, heavy academic pressure, drastic change in learning mode under the pandemic, and increased insecurity about career prospect under the pandemic (Liyanage et al., 2021; Shek et al., 2022a).

Unfortunately, existing research on the mental health of university students during the pandemic and its risk and protective factors is limited. Besides, most of the existing studies were based on university students in Western countries (Wang et al., 2020; Liyanage et al., 2021), which may not capture the behavior of university students in non-Western contexts such as Hong Kong. As a previous city under colonial rule of the United Kingdom and a current special administrative region of China, Hong Kong has experienced various challenges in recent years including social movements and the pandemic. Under this situation, the mental health of university students in Hong Kong might face more challenges. Several existing studies showed the high prevalence of mental health problems in university students in Hong Kong in recent years and under the pandemic (Shek et al., 2022a,b, 2023a,b). However, the potential risk and protective factors for the mental health of university students under the pandemic still remain unclear and not fully examined. Research in this study does not only contribute to theoretical advancement but also has practical implications. Particularly, effective university policymaking and the development of effective prevention and intervention programs need a comprehensive understanding of the potential factors that may either increase or lower the risk for mental health problems in university students in Hong Kong under the pandemic.

To gain a comprehensive understanding of the issue, this study adopted an integrated model incorporating the ecological systems theory and the perspective of positive youth development (PYD) to guide this study (Shek et al., 2023b) to explore the correlates of Hong Kong university students’ mental health under the pandemic. In accordance with the ecological systems theory (e.g., Bronfenbrenner, 2001), there are inter-connected personal and environmental systems shaping adolescent development. From the PYD perspective, the internal and external developmental assets help to “protect” young people when facing life challenges (Scales et al., 2000). Benson (2007) proposed 20 internal assets (e.g., psychosocial competence and optimism) and 20 external assets (e.g., support from family) which shape and contribute to the positive development of youth and adolescents (Morales-Rodríguez et al., 2020; Shek et al., 2022a). In this study, we conceived mental health with regard to psychological morbidity (e.g., psychological symptoms such as anxiety, depression, and Internet addiction) and positive well-being (e.g., flourishing and satisfaction with life). This conception of mental health is widely adopted in many studies in public health (Shek et al., 2022a, 2023b). We focused on four groups of correlates of mental health, including COVID-19 related stress factors, intrapersonal factors, ecological factors, and factors on need satisfaction, difficulties encountered, and university services. Below is a detailed account of the role of these factors based on literature.

First, as mentioned earlier, as a global public health crisis, COVID-19 may trigger high levels of stress, threats, and worries in the general population, including university students, due to its high death rates, high contagiousness, and related social and economic consequences caused by lockdown and social distancing policies. These worries, stresses, and fears may trigger dysfunctional cognitive-behavioral coping strategies to respond to the threat of the pandemic (Mansueto et al., 2022; Alhakami et al., 2023), which then lead to increased mental health problems and psychological distress (Kennedy et al., 2021; Magson et al., 2021). Hence, it is important to investigate the role of COVID-19 stress on university students’ psychological well-being. Although recent studies (Zolotov et al., 2020; Shek et al., 2023b) identified that COVID-19 associated stress was positively connected to psychological morbidity and negatively associated with positive well-being, it is necessary to replicate these findings at a different stage of the pandemic and with a different sample.

Besides COVID-19 stress, different internal development assets such as resilience, emotional competence, and positive beliefs about adversity also play an important role in university students’ mental health. Regarding the role of resilience, studies showed that resilience buffered the negative influence of stress in psychological crises on mental health, enabling individuals to adapt successfully to adversity (Masten et al., 2021). University students with stronger resilience also showed better developmental outcomes (Hartley, 2011; Wu et al., 2020), especially under the pandemic period (Shek et al., 2022a, 2023b). Emotional competence is another internal asset associated with psychological well-being in stressful situations (Di Fabio and Kenny, 2016). Recent research showed that emotional competence predicted lower levels of fear, anxiety, and depression under COVID-19 (Moroń and Biolik-Moroń, 2021), including university students (García-Álvarez et al., 2021). Finally, studies have highlighted the influence of positive perceptions of adversities on psychological well-being and life satisfaction. For example, Wang and Liu (2022) highlighted the importance of positive belief about adversity in reducing depressive symptoms in Chinese adolescents. In addition, Shek et al. (2023a) showed that beliefs about adversity was a negative predictor of psychological morbidity but a positive predictor of positive well-being.

Besides factors in the intrapersonal system such as psychosocial competence, factors in the interpersonal context such as family relationship also play an important role in student psychological well-being (Magson et al., 2021). In particular, the family system holds a particularly significant role in adolescents’ development (Albanese et al., 2019). While a supportive family environment creates a safe and harmonious space that can significantly impact children’s help-seeking behavior and their ability to find effective solutions to their psychological problems (Ebrahimi et al., 2019; Wiguna et al., 2020), negative characteristics of the family environment, such as conflict, have been linked to adverse outcomes for adolescents (Ward and Lee, 2020). During the pandemic, adolescents have been confined to their homes, leading to increased levels of stress and anxiety, possibly due to greater family conflict (Zeng et al., 2021). At the same time, mutuality within the family may increase under the pandemic. Hence, it is important to examine both positive and negative aspects of family functioning.

Besides internal and external developmental assets, need satisfaction and encountered difficulties also predict the mental health of university students (Shek et al., 2022a, Forthcoming). The self-determination theory and related research suggest that the degree to which individuals’ needs are met can significantly impact their capacity to adjust to challenging circumstances (Deci and Ryan, 2008; Schutte and Malouff, 2021), including the pandemic context (Šakan et al., 2020; Zhou et al., 2020). However, under the pandemic, the fulfillment of university students’ needs may be adversely affected, and they face challenges in life (Shek et al., 2022b). At the university level, student mental health has been linked to various aspects of university support, including connectedness, atmosphere, engagement, counseling services, accessibility to support, financial aid, and career development services (Oldfield et al., 2018). Hence, effective support and services provided by universities act as protective factors. For example, A study showed that university support negatively predicted mental health problems in college students (Legros and Boyraz, 2023). Brenes et al. (2015) also indicated the effectiveness of tele-mental health service in treating anxiety and depressive symptoms. Hence, it is important to understand how need satisfaction, difficulties encountered, and factors related to university services predict psychological well-being.

Although existing research has demonstrated roles of different correlates of university students’ psychological well-being during the pandemic, there are several gaps in the literature that warrant further investigation. First, few studies have employed a comprehensive framework that incorporates different intrapersonal and interpersonal factors influencing student well-being. Second, few studies have included different measures of mental health in a single study. Third, despite universities’ crucial role in satisfying students’ needs under COVID-19, limited studies have explored how their needs, difficulties encountered, and university services are related to their well-being. Fourth, although some previous studies have addressed the questions included in this study, they were conducted at Wave 4 (Shek et al., 2022a) and Wave 5 (Shek et al., 2023b) of the pandemic. Hence, it is necessary to examine the issues at a time when the pandemic eases. Fifth, to replicate the findings reported previously, there is a need to examine the related questions in freshmen who also face additional burdens arising from transition from high school to university education.

Finally, whether there are gender differences in the predictive effects of different factors on mental health of university students remains unclear. Some studies suggest that female students might be more vulnerable to mental health problems in stressful situation as they are more likely to have low sense of mastery and negative coping than male students due to both biological and cultural mechanisms (Nolen-Hoeksema et al., 1999; Cao, 2022). This might be more obvious in Chinese society as the image of women in traditional Chinese culture is featured by “tenderness” and “introversion” while the image of men is characterized by “self-confidence” and “sense of responsibility” (Cao, 2022, p. 4). However, there are also studies showing no gender difference in the relationship between negative life events or other intra- and inter-personal predictors (such as emotional intelligence) and mental health problems (Sergi et al., 2021). Therefore, it is necessary to explore this issue further.

To address the above-mentioned research gaps, this study examined the status of mental health symptoms and well-being of university students in the pandemic period, as well as the psychosocial correlates. We raised six research questions below:

Research Question 1: What are the prevalence rates of psychological morbidity among Hong Kong university students? Based on previous two studies conducted based on Hong Kong college students (Shek et al., 2022b, 2023b) and elsewhere (Czeisler, 2020), we hypothesized that there would be higher prevalence rates of mental health morbidity, such as anxiety, stress, and depressive symptoms in university students under the COVID-19 pandemic (Hypothesis 1).

Research Question 2: Is COVID-19 associated stress related to negative mental health and positive well-being in students in universities in Hong Kong? Referring to previous studies (Zolotov et al., 2020; Shek et al., 2023b), we hypothesized that different types of COVID-19 related stress would be related to psychological morbidity in a positive direction (Hypothesis 2a) and related to positive well-being in a negative direction (Hypothesis 2b).

Research Question 3: What is the relationship between intrapersonal competencies and university students’ psychological well-being? Based on previous findings (Masten et al., 2021; Shek et al., 2022a,b), we hypothesized that intrapersonal factors (indexed by beliefs about adversity, resilience, and emotional competence) would be related to psychological morbidity negatively (Hypothesis 3a) and associated with well-being positively (Hypothesis 3b).

Research Question 4: Does family functioning predict university students’ psychological well-being? Referring to existing research findings (Ebrahimi et al., 2019), we hypothesized that a positive family environment would have negative association with psychological morbidity (Hypothesis 4a) and positive association with well-being (Hypothesis 4b), while negative family functioning would have positive association with mental health issues (Hypothesis 4c) and negative correlation with well-being (Hypothesis 4d).

Research Question 5: Is satisfaction of needs, difficulties encountered, and students’ perceptions of university services related to student mental health? Based on related theories (Wang et al., 2020) and research findings (Shek et al., 2022b, 2023b), we hypothesized that satisfaction of needs, students’ knowledge, positive perceptions and evaluation of university services would be linked positively to their well-being (Hypothesis 5a) and correlated negatively with their psychological morbidity (Hypothesis 5b). Conversely, perceived difficulties would be negatively related to positive well-being (Hypothesis 5c) and positively related to psychological morbidity (Hypothesis 5d) among university students.

Research Question 6: Whether there are gender differences in the relationships between predictors in different ecological systems and mental health of university students? Due to inconclusive findings in the existing literature (Sergi et al., 2021; Cao, 2022), we did not have hypothesis for this research question.

## 2 Materials and methods

### 2.1 Participants and procedures

Quota sampling was used in this study which is a non-probability sampling method selecting participants from a population based on certain stratifying characteristics (Rukmana, 2014). We recruited participants from the first-year undergraduate students in one public university in Hong Kong with faculty as a stratifying characteristic. The participants were invited to complete a survey questionnaire through a web-based survey platform Qualtrics XM during the late stage of COVID-19 epidemic in Hong Kong (October 2022–February 2023). Since October 2022, the Hong Kong government has gradually relaxed and lifted its anti-epidemic measures. Then the government has canceled all social distancing measures and relaxed other epidemic prevention policies in late December of 2022 and the WHO has announced that the COVID-19 has no longer constituted a public health emergency in early May of 2023 (The Government of the Hong Kong Special Administrative Region, 2023). In total, 1,020 first-year undergraduate students (mean age = 18.70 ± 1.46) completed the questionnaire (see Table 1). Before the survey, ethic approval was granted by the institutional ethics review board of the university. Students gave formal informed consent before joining the study.

**Table 1:** Socio-demographic characteristics of the participants in the final sample (*N* = 1,020).

Variable	***n***	%
Faculty		
Faculty of engineering	211	20.7
Faculty of construction and environment	98	9.6
Faculty of health and social sciences	396	38.8
Faculty of applied science and textile	67	6.6
Faculty of humanities	39	3.8
Faculty of business	93	9.1
School of design	42	4.1
School of hotel and tourism management	43	4.2
School of fashion and textiles	31	3.0
Gender		
Male	451	44.2
Female	517	50.7
Prefer not to say	52	5.1
Received CSSA		
Yes	902	88.4
No	40	3.9
Not sure	78	7.6
Experienced financial difficulty (family)		
Yes	684	67.1
No	184	18.0
Not sure	152	14.9
Experienced financial difficulty (personal)		
Yes	682	66.9
No	248	24.3
Not sure	90	8.8
Student or their family members unemployed during the pandemic		
Yes	810	79.4
No	145	14.2
Not sure	65	6.4
Student had been a confirmed case of COVID-19		
Yes	508	49.8
No	475	46.6
Not sure	37	3.6
Family members had been a confirmed case of COVID-19		
Yes	369	36.2
No	600	58.8
Not sure	51	5.0
Living status		
Live with family	857	84.0
Live with roommates	149	14.6
Live alone	14	1.4
Place of residence during the pandemic		
Hong Kong	987	96.8
Mainland China	29	2.8
Others	4	0.4
Place of origin (local/international student)		
Local	858	84.1
International	162	15.9

CSSA, comprehensive social security assistance scheme.

### 2.2 Measures

#### 2.2.1 Measures of COVID-19 stress

The “COVID Stress Scale” developed by Taylor and his colleagues (Taylor et al., 2020) was adopted as the measure of university students’ perceived stress related to the pandemic. This measure contains three dimensions (five items for each dimension), including “the danger and contamination of COVID-19″ (DC), “the socio-economic consequences of COVID-19″ (SC), and “check behavior caused by COVID-19 related concerns” (CB). The participants rated how often the listed situations occurred to them on a scale from “0″ = *“Not at all”* to “4″ = *“Always.”* The COVID stress was represented by the mean score of items. Previous studies showed that this scale possessed adequate psychometric properties (Shek et al., 2022a, 2023b).

#### 2.2.2 Measures of psychological morbidity

##### 2.2.2.1 Depression anxiety stress scale

The participants’ experiences of three major mental health problems: anxiety, stress, and depression, were gauged by the “Depression Anxiety Stress Scale (DASS-21).” The measure includes 21 items with each mental health problem being measured by one subscale (seven items). The participants rated how frequently they have experienced each listed symptom on a measure (“0″ = *“Not at all”* to “3″ = *“Most of the time”*). The item total score in each subscale was calculated to indicate the three mental health problems. The psychometric properties of the measure have been examined by previous research (Zanon et al., 2021).

##### 2.2.2.2 The center for epidemiologic studies depression scale revised

The “Center for Epidemiologic Studies Depression Scale Revised (CESD-R)” (20 items) was adopted to measure depressive disorder referring to “The Diagnostic and Statistical Manual of Mental Disorders, Fifth Edition (DSM-V)” (Radloff, 1977; Eaton et al., 2004). The students reported how frequently they have displayed the symptom described in each item through a rating measure (“0” = *“Not at all or less than 1 day”* to “4” = *“Nearly every day for 2 weeks”*). The item total score represents the level of depressive symptoms. The psychometric properties of the measure were examined and established by previous studies (Van Dam and Earleywine, 2011; Shek et al., 2023b).

##### 2.2.2.3 Young’s 10-item internet addiction test

“Young’s 10-item Internet Addiction Test (IAT-10)” (Young, 1998) was used as the scale of Internet addiction (IA) in this study. This measure consists of 10 items assessing the addictive symptoms regarding Internet use (Pawlikowski et al., 2013). The Chinese version of the IAT-10 was also validated by Shek and his colleagues (Shek et al., 2008). The students indicated whether they had suffered from the listed symptoms over the past 12 months by choosing “1” = *“Yes”* or “0” = *“No.”* The IA level was indicated by the sum score of all items.

##### 2.2.2.4 Suicidal behavior scale

“The Suicidal Behavior Scale” comprised of 3 questions asking students’ plans, attempts, and thoughts regarding committing suicide over the past 12 months was adopted to measure suicidal behavior in this study. The participants responded to each question by choosing “1″ = *“Yes”* or “0″ = *“No”* (Shek et al., 2023b). The item total score represents the score of suicidal behavior. Previous studies (e.g., Shek et al., 2022a, 2023b) suggest acceptable psychometric properties of the measure.

##### 2.2.2.5 Hopelessness scale

“The Chinese Hopelessness Scale (CHOPE)” (Shek, 1993) was adopted to assess hopelessness of university students in this study. This measure consists of five items assessing individuals’ hopelessness about their lives. The students indicated to what extent they agreed with each description via a rating measure (“1″ = *“Strongly disagree”* to “6″ = *“Strongly agree”*). The average of all item scores was calculated as the indicator of hopelessness in the present study. The psychometric properties of CHOPE were tested by previous studies (Shek et al., 2022a, 2023b).

#### 2.2.3 Measures of positive well-being

##### 2.2.3.1 The satisfaction with life scale

University students’ satisfaction with life was gauged by “The Satisfaction with Life Scale (SWLS)” (Diener et al., 1985), which includes five items. Participants reported their level of satisfaction with life via a rating measure (“1″ = *“Strongly disagree”* to “6″ = *“Strongly agree”*). The scale score was indicated by the item average score. Previous studies showed desirable psychometric properties of SWLS (Wu et al., 2009; Bai et al., 2010).

##### 2.2.3.2 Flourishing scale

The “Flourishing Scale (FS)” was used to measure participants’ perceived flourishing in their lives. This scale includes eight items regarding individuals’ psychological well-being in different aspects of their lives (Diener et al., 2010). Participants reported their agreement with the listed description in each item via a rating measure (“1″ = *“Strongly disagree”* to “7″ = *“Strongly agree”*). The mean score for all items was calculated as the indicator of flourishing in this study. The psychometric properties of FS have been tested by previous studies.

#### 2.2.4 Measures of intrapersonal correlates

##### 2.2.4.1 Cultural beliefs about adversity scale

The participants’ adversity related beliefs were assessed using “The Chinese Cultural Beliefs about Adversity Scale (CBA)” (Shek et al., 2003). The CBA has nine items, among which seven items measure participants’ agreement with seven positive traditional Chinese sayings on adversity and two items (reverse coded) measure their agreement with two negative traditional Chinese sayings on adversity. The two reverse coded items were removed from the scale due to criticism of their effectiveness in different studies (Sonderen et al., 2013) and undesirable factor loadings in the SEM model of this study. The participants provided their answers on a rating measure (from “1″ = *“Strongly disagree”* to “6″ = *“Strongly agree”*). The item mean score represents the levels of beliefs of adversity. Previous research showed that this scale possessed adequate psychometric properties (Shek et al., 2022a, 2023b).

##### 2.2.4.2 Chinese positive youth development scale

Two subscales from the “Chinese Positive Youth Development Scale (CPYDS)” (Shek et al., 2007) were adopted to evaluate the emotional competence and resilience of the students, with each subscale including three items. The students indicated to what degree they agree with the description of themselves in each statement through a rating measure (“1” = *“Strongly disagree”* to “6” = *“Strongly agree”*). The two interpersonal competencies were represented through the mean scores of respective subscale items. Previous research suggests that the measure possessed adequate psychometric properties (Shek et al., 2022a, 2023b).

#### 2.2.5 Measure of interpersonal correlates

##### 2.2.5.1 Family functioning

Family functioning was examined through the “Chinese Family Assessment Instrument (C-FAI)” (Shek, 2002). These included positive functioning of family (“Family Communication” and “Family Mutuality”) and negative functioning of family indexed by “Family Conflicts,” with each subscale including 3 items. However, due to undesirable factor loading of one item in Family Conflicts in the SEM model of this study, the item was removed from the scale. The participants evaluated the extent the described situation in each item was consistent with that in their own families through a five-point measure (“1” = *“Very unlike”* to “5” = *“Very like”*). Positive family functioning was represented by the average score of items in the two positive subscales, and negative family functioning was represented by the average score of the items in the negative sub-scale.

#### 2.2.6 Measures of perceived satisfaction of needs, difficulties, and university services

##### 2.2.6.1 Need satisfaction

University students’ need satisfaction under COVID-19 was examined by a 15-item measure. The measure evaluates the extent to which university students’ needs in various life domains have been met during the pandemic, which was developed based on student focus groups by the authors of the study. The participants reported their answers through a rating measure (“1″ = *“not met at all”* to “6″ = *“fully met”*). The item mean score was adopted as the composite score (see Shek et al., 2023b). The scale possessed good reliability in previous research (Shek et al., 2022b).

##### 2.2.6.2 Difficulties encountered

A 24-item self-developed measure was used to gauge the challenges and difficulties perceived by students during the pandemic. Students reported how often they encountered the difficulties through a five-point measure (“1” = *“Never”* to “5” = *“Always”*). An average score was used indicating a composite score. Previous research reported good reliability of the measure (Shek et al., 2022b).

##### 2.2.6.3 Knowledge and perception of usefulness of university service

Students’ knowledge of university services during COVID-19 (e.g., “Counselling and Wellness Section,” “University Health Service,” and “Special Funding under COVID-19″) was measured using a 10-item binary scale. Students indicated whether they knew about the services listed (“1″ = *“yes”* and “0″ = *“no”*). The measure score was calculated through summing all item scores. Besides, students’ views of usefulness of the aforementioned services were measured through a 10-item self-developed measure. Students rated their perceptions of usefulness of the services via a six-point measure (“1″ = *“Not at all”* to “6″ = *“Completely yes”*). The variable score was calculated through averaging the items scores.

##### 2.2.6.4 Evaluation of university service

Another self-developed measure was adopted assessing students’ evaluation of service/support offered by the university during the pandemic. With eight items, students rated to what degree they were satisfied with different services offered by the university related to the pandemic via a rating measure (“1″ = *“Strongly disagree”* to “6″ = *“Strongly agree”*). The mean score was calculated to represent students’ subjective evaluation of university services.

### 2.3 Data analysis

First, we conducted reliability and descriptive analyses on all key constructs. Second, the prevalence of mental health symptoms (indicated by stress, anxiety, and depression [DASS-21], depressive symptoms [CESD-R], suicidal behavior [SBS], and Internet addiction) was presented. Third, Pearson’s correlation with Bonferroni correction was conducted between all key variables. As the total number of correlations is 190, the *p* value has been adjusted to 0.05/190 = 0.0003. Fourth, structural equation models (SEM) on the predictive effects of stress related to COVID (Model 1), intrapersonal competence (Model 2), family functioning (Model 3), and needs, difficulties, and service-related factors (Model 4) on mental health symptoms and positive well-being were tested (Shek et al., 2023b; Shek et al., in press). Finally, multigroup SEM was conducted to examine gender differences in the four SEM models, which included two steps. In the first step, the scalar invariance across groups in the measurement model of each SEM model was examined as the prerequisite for multigroup SEM (Wolgast and Donat, 2019; Vinter et al., 2021). On the condition of establishment of scalar invariance, multigroup SEM was then conducted through comparing the configural model and a model with path coefficients in both groups being constrained to be equal. Multiple indices including “comparative fit index” (CFI), “Tucker-Lewis index” (TLI), “the root mean square error of approximation” (RMSEA), and “standardized root mean square residual” (SRMR) were used to evaluate model fit, with CFI and TLI > 0.90 (Kline, 2023) and RMSEA and SRMR <0.08 (Browne and Cudeck, 1993) indicating desirable model fit. For model comparison, as chi-square test is sensitive to large sample size, change in CFI (∆CFI > 0.01) (Cheung and Rensvold, 2002) was used as criteria for judging whether the two models (configural model vs. constrained model) have significant difference. All the SEM analyses were conducted using R 4.2.1 with the use of robust maximum likelihood estimation (MLR).

## 3 Results

### 3.1 Descriptive profile and measure reliability

Results of reliability and descriptive analyses are shown in Table 2. All measures showed acceptable internal consistency (*α* = 0.60–0.96) and the mean inter-item correlations was between 0.26 and 0.71.

**Table 2:** Reliability, mean, and standard deviation of measures of different variables.

Measure	Cronbach’*α*	Inter-item correlation	*M*	*SD*
Negative mental health				
Depression anxiety stress scale (DASS-21)	0.94	0.44	0.80	0.52
Center for epidemiologic studies depression scale revised (CESD-R)	0.95	0.48	15.26	12.84
Young’s 10-item internet addiction test (IAT-10)	0.83	0.32	3.60	2.91
Suicidal behavior scale (SBS)	0.60	0.41	0.27	0.62
Chinese hopelessness scale (CHOPE)	0.87	0.57	3.12	0.96
Positive wellbeing				
The satisfaction with life scale (SWLS)	0.88	0.60	3.58	1.02
The flourishing scale (FS)	0.93	0.61	4.76	1.09
COVID-19 related factors				
The COVID stress scale				
Danger and contamination subscale (DC)	0.91	0.66	1.39	0.95
Socio-economic consequences subscale (SC)	0.87	0.60	0.82	0.79
Checking behavior subscale (CB)	0.85	0.53	1.30	0.81
Intrapersonal factors				
Chinese cultural beliefs about adversity scale (CBAS)	0.87	0.48	4.33	0.84
Chinese positive youth development scale (CPYDS)				
Resilience subscale	0.86	0.68	4.26	0.88
Emotional competence subscale	0.80	0.55	4.23	0.87
Ecological factors				
Chinese family assessment instrument (C-FAI)				
Positive family functioning	0.91	0.62	3.65	0.85
Negative family functioning	0.71	0.56	2.80	1.00
Needs, difficulties, and services				
Difficulties encountered	0.90	0.35	2.69	0.35
Needs satisfaction	0.92	0.43	4.19	0.82
Knowledge of service	0.78	0.26	5.30	2.67
Usefulness of service	0.96	0.71	4.28	0.99
Evaluation of service	0.93	0.62	4.10	0.83

### 3.2 Prevalence of psychological morbidity

Table 3 shows the prevalence of psychological morbidity in the participants. Based on the DASS-21 criteria, 60.8, 54, and 33.2% of the participants showed mild to extremely severe levels of anxiety, depression, and stress, respectively. Regarding CESD-R, 41.6% of the participants were identified as experiencing depression (scored 16 or above) (Radloff, 1977). Besides, based on IAT-10 criteria (Pawlikowski et al., 2013), 47.3% of the students showed internet addiction problems (4 or more symptoms). In addition, 18.2% of the students indicated having suicidal thoughts, and 5.4 and 3.4% of the participants reported having suicidal plans and suicidal attempts, respectively. These findings generally supported Hypothesis 1.

**Table 3:** Prevalence of negative mental health.

Measure and category	*n*	%
DASS-21-depression^a^		
Normal	469	46.0
Mild	173	17.0
Moderate	252	24.7
Severe	73	7.2
Extremely severe	53	5.2
DASS-21-anxiety^b^		
Normal	400	39.2
Mild	86	8.4
Moderate	304	29.8
Severe	94	9.2
Extremely severe	136	13.3
DASS-21-stress^c^		
Normal	681	66.8
Mild	150	14.7
Moderate	117	11.5
Severe	57	5.6
Extremely severe	15	1.5
CESD-R		
With symptoms (scored 16 or above)	424	41.6
Without symptoms	596	58.4
IAT-10		
Addicted (scored 4 or above)	482	47.3
Not addicted	538	52.7
SBS		
Have suicidal thoughts	186	18.2
Have suicidal plans	55	5.4
Have suicidal attempts	35	3.4
CHOPE		
Negative direction (mean score ≥ 4)	218	21.4

^a^Depression: normal = 0–9, Mild = 10–13, Moderate = 14–20, Severe = 21–27, Extremely severe = 28 or above. ^b^Anxiety: Normal = 0–7, Mild = 8–9, Moderate = 10–14, Severe = 15–19, Extremely severe = 20 or above. ^c^Stress: Normal = 0–14, Mild = 15–18, Moderate = 19–25, Severe = 26–33, Extremely severe = 34 or above.

### 3.3 Psychosocial correlates of psychological morbidity and positive well-being

**Figure 1:**
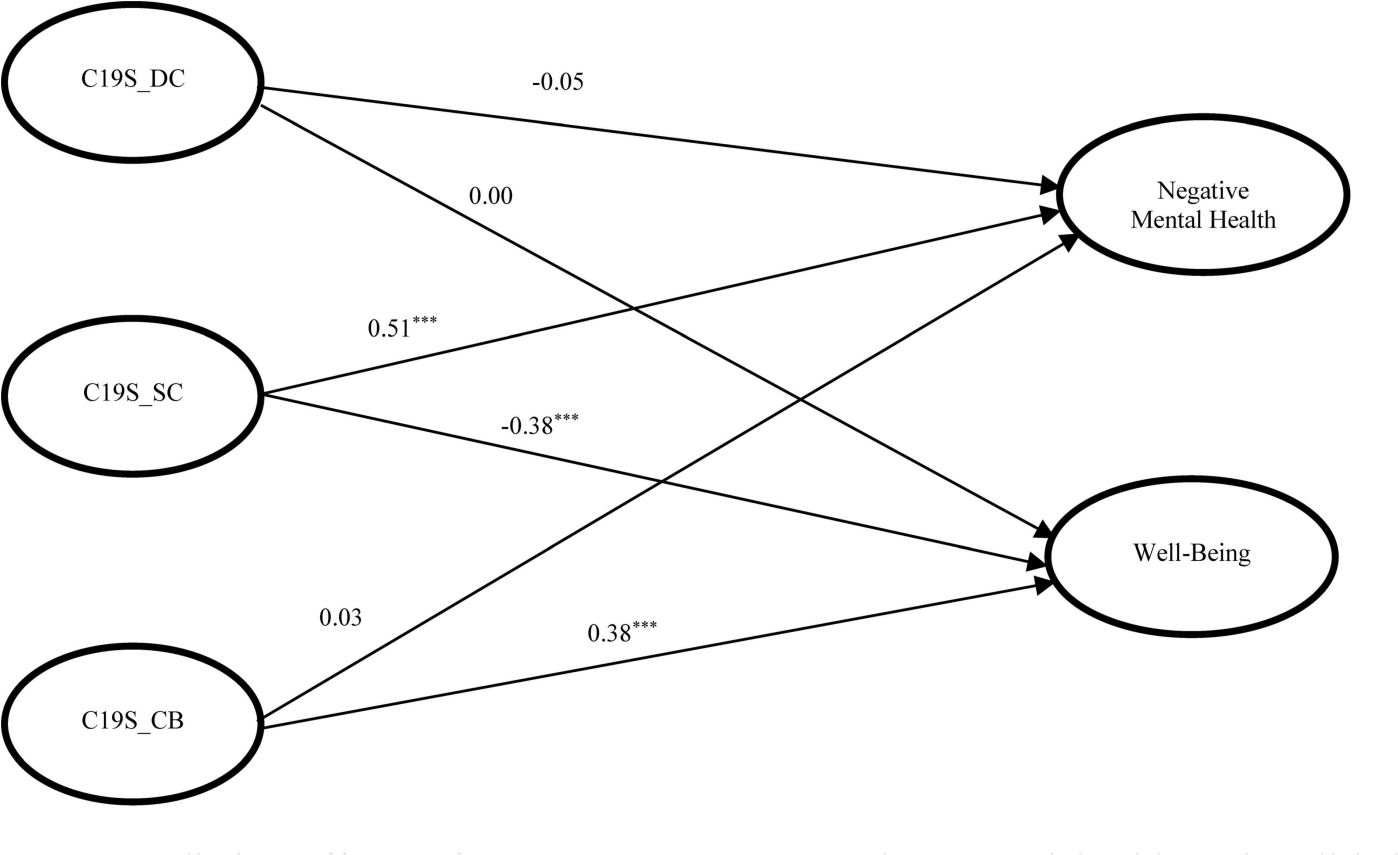
Predictive effects of COVID stress on negative mental health and well-being (Model 1). *x^2^/df* = 4.32, CFI = 0.931, TLI = 0.920, RMSEA = 0.057, SRMR = 0.050; C19S, COVID-19 related stress; DC, danger and contamination; SC, socio-economic consequences; CB, check behavior; ^***^p < 0.001.

**Figure 2:**
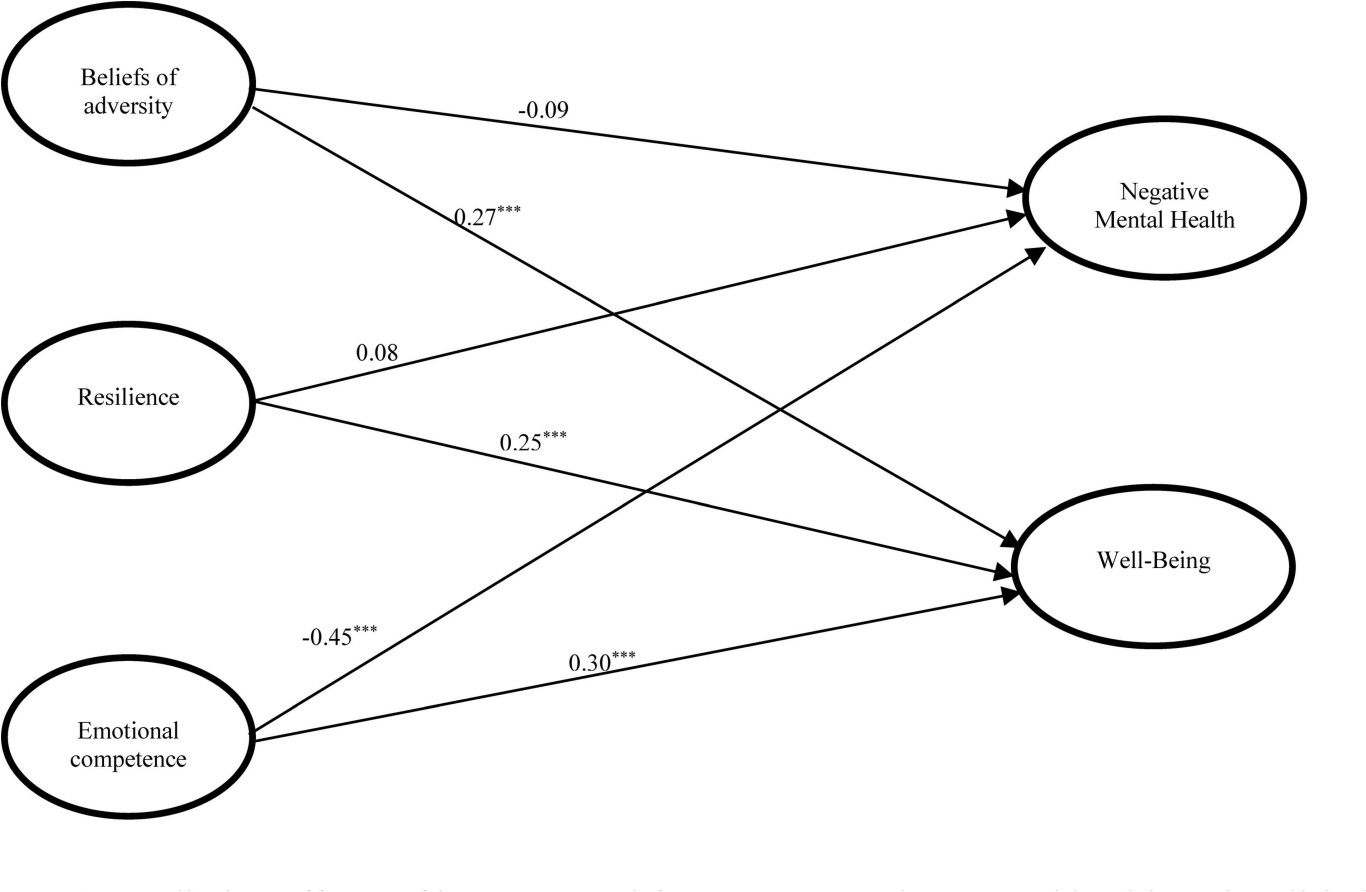
Predictive effects of intrapersonal factors on negative mental health and well-being (Model 2). *x^2^/df* = 3.51, CFI = 0.944, TLI = 0.934, RMSEA = 0.050, SRMR = 0.047; ^***^ *p < 0.001*.

**Figure 3:**
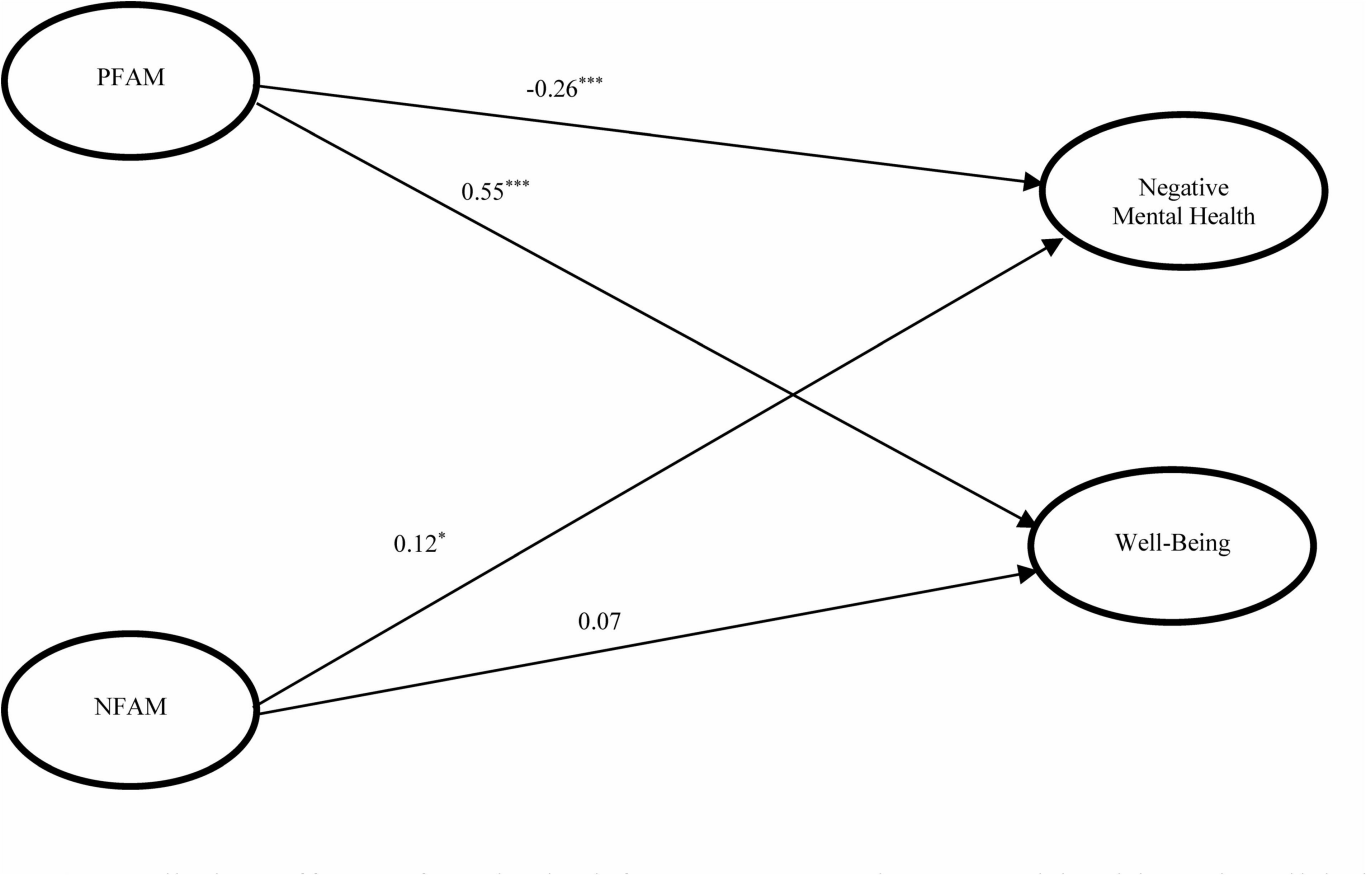
Predictive effects of ecological factors on negative mental health and well-being (Model 3). *x^2^/df* = 4.83, CFI = 0.944, TLI = 0.930, RMSEA = 0.061, SRMR = 0.052; PFAM, positive family functioning; NFAM, negative family functioning; ^***^p < 0.001; ^*^p < 0.05.

**Figure 4:**
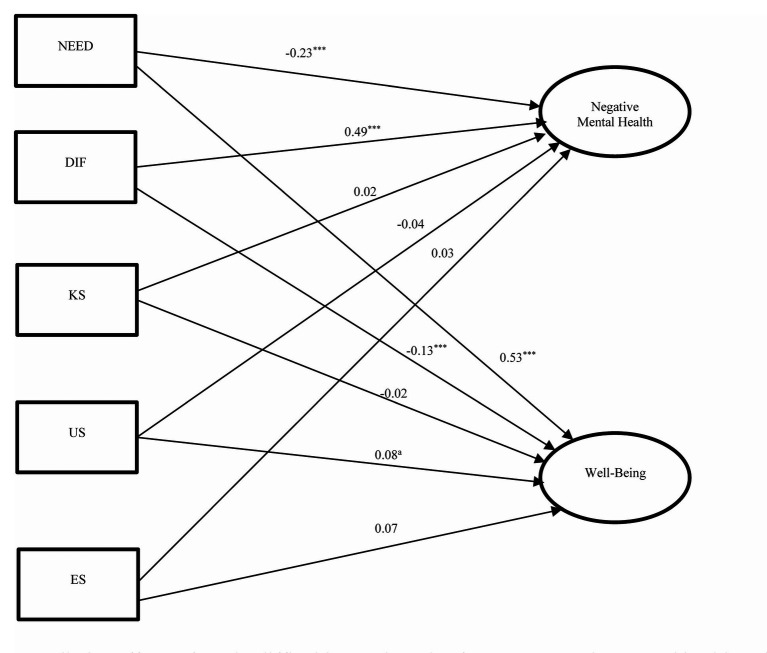
Predictive effects of needs, difficulties, and service factors on negative mental health and well-being (Model 4). *x^2^/df* = 5.33, CFI = 0.932, TLI = 0.900, RMSEA = 0.067, SRMR = 0.047; NEED, needs satisfaction; DIF, difficulties encountered; KS, knowledge of university services; US, perceived usefulness of university services; ES, evaluation of university services; ms, marginal significance (*p* = 0.062); ^*^p < 0.05; ^***^p < 0.001, ^a^*p* = 0.05.

Table 4 demonstrates the results of correlation analyses among major variables. Generally speaking, the variables were correlated in the hypothesized directions. Figures 1–4 illustrate the results of the SEM analyses (Model 1 to 4) on the predictive effects of psychosocial factors on psychological morbidity and positive well-being. In Model 1, three latent variables of COVID-19 stress, including DC, SC, and CB were used. In Model 2, three latent factors (beliefs of adversity, resilience, and emotional competence) were included. In Model 3, latent variables of positive family functioning and negative family functioning were the predictors. For Model 4, we used the total score of each scale to separately reflect need satisfaction, difficulties, and service-related factors as there are large number of items in these variables (a total of 59 items) which will increase model complexity and cause difficulty for model fit if using latent variables of these predictors (Moshagen, 2012; Cao and Liang, 2022). For outcome variables, negative mental health was indexed by the total scores of DASS-21 (depression, anxiety, and stress), CESD-R (symptoms of major depressive disorder), IAT-10 (internet addiction), and SBS (suicidal behavior), and the mean score of CHOPE (hopelessness). For positive well-being, it was indicated by the mean scores of SWLS (life satisfaction) and FS (flourishing) in all the four models.

**Table 4:** Correlations among key variables.

Variable	1	2	3	4	5	6	7	8	9	10	11	12	13	14	15	16	17	18	19
1. CS_DC	--																		
2. CS_SC	0.50^***^	--																	
3. CS_CB	0.58^***^	0.57^***^	--																
4. DASS	0.18^***^	0.40^***^	0.24^***^	--															
5. CESDR	0.23^***^	0.45^***^	0.27^***^	0.79^***^	--														
6. SB	0.01	0.13^***^	0.06	0.31^***^	0.34^***^	--													
7. IA	0.16^***^	0.22^***^	0.16^***^	0.36^***^	0.41^***^	0.32^***^	--												
8. HL	0.10	0.32^***^	0.13^***^	0.44^***^	0.45^***^	0.21^***^	0.24^***^	--											
9. LS	0.09	0.01	0.17^***^	−0.33^***^	−0.29^***^	−0.22^***^	−0.23^***^	−0.24^***^	--										
10. FH	0.02	−0.14^***^	0.10	−0.42^***^	−0.38^***^	−0.23^***^	−0.23^***^	−0.38^***^	0.68^***^	--									
11. BA	0.07	−0.10	0.06	−0.25^***^	−0.24^***^	−0.17^***^	−0.12^***^	−0.23^***^	0.38^***^	0.60^***^	--								
12. RE	0.10	−0.11^***^	0.07^*^	−0.28^***^	−0.24^***^	−0.18^***^	−0.15^***^	−0.30^***^	0.43^***^	0.62^***^	0.63^***^	--							
13. EC	−0.01	−0.15^***^	−0.01	−0.34^***^	−0.32^***^	−0.13^***^	−0.22^***^	−0.23^***^	0.38^***^	0.59^***^	0.53^***^	0.65^***^	--						
14. PFAM	0.04	−0.09	0.06	−0.26^***^	−0.24^***^	−0.20^***^	−0.11^***^	−0.29^***^	0.37^***^	0.46^***^	0.43^***^	0.41^***^	0.39^***^	--					
15. NFAM	0.05	0.12^***^	0.04	0.16^***^	0.16^***^	0.13^***^	0.02	0.27^***^	−0.09	−0.10	−0.06	−0.09	−0.05	−0.31^***^	--				
16. DIF	0.28^***^	0.40^***^	0.26^***^	0.46^***^	0.49^***^	0.19^***^	0.31^***^	0.54^***^	−0.25^***^	−0.31^***^	−0.19^***^	−0.20^***^	−0.22^***^	−0.27^***^	0.19^***^	--			
17. NEED	0.05	−0.09^**^	0.10	−0.32^***^	−0.29^***^	−0.21^***^	−0.21^***^	−0.29^***^	0.43^***^	0.58^***^	0.51^***^	0.50^***^	0.48^***^	0.55^***^	−0.09	−0.32^***^	--		
18. KS	−0.03	−0.12^***^	−0.01	−0.11^***^	−0.14^***^	0.05	0.00	−0.19^***^	0.07	0.14^***^	0.14^***^	0.09^**^	0.14^***^	0.17^***^	−0.08	−0.23^***^	0.19^***^	--	
19. US	0.11^***^	−0.08	0.15^***^	−0.16^***^	−0.13^***^	−0.12^***^	−0.02	−0.17^***^	0.19^***^	0.31^***^	0.33^***^	0.35^***^	0.27^***^	0.27^***^	−0.12	−0.11	0.39^***^	−0.04	--
20. ES	0.02	−0.08^*^	0.13^***^	−0.24^***^	−0.20^***^	−0.15^***^	−0.15^***^	−0.28^***^	0.32^***^	0.44^***^	0.40^***^	0.42^***^	0.40^***^	0.37^***^	0.01	−0.30^***^	0.55^***^	0.25^***^	0.46^***^

CS, COVID stress; DC, anger and contamination; SC, socio-economic consequences; CB, check behavior; DASS, the total score of depression, anxiety stress scale; CSSD-R, the center for epidemiologic studies depression scale revised, SB, suicidal behavior; IA, internet addiction; HL, hopelessness; LS, life satisfaction; FL, flourishing; BA, beliefs of adversity; RE, resilience; EC, emotional competence; PFAM, positive family functioning; NFAM, negative family functioning; NEED, needs satisfaction; DIF, difficulties encountered; KS, knowledge of university services; US, perceived usefulness of university services; ES, evaluation of university services; ^***^p < 0.0003 (the significance level has been adjusted to 0.0003 based on Bonferroni correction).

All the four SEM models yielded good model fit with χ2/df values ranging between 3.51 to 5.33, CFI and TLI values being above 0.90 and RMSEA and SRMR values being below 0.08 (Table 5). For all the models, the factor loadings of observed variables for different latent variables ranged between 0.38 and 0.99. Figure 1 shows the predictive effects of the three components of COVID-19 stress (DC, SC, and CB) on mental health illness and positive well-being (Model 1). While the predictive effects of DC were not significant on both negative and positive mental health, SC positively predicted negative mental health (*β* = 0.51, *p* < 0.001) and negatively predict well-being (*β* = −0.38, *p* < 0.001). CB had no predictive effect on mental health illness but predicted well-being in a positive direction (*β* = 0.38, *p* < 0.001). Hypotheses 2a and 2b were partially supported.

**Table 5:** Model fix indices of the four SEM models.

Model	*χ^2^*/*df*	CFI	TLI	RMSEA	SRMR
Model 1: predictive effects of COVID stress	4.32	0.931	0.920	0.057	0.050
Model 2: predictive effects of intrapersonal factors	3.51	0.944	0.934	0.050	0.047
Model 3: predictive effects of ecological factors	4.83	0.944	0.930	0.061	0.052
Model 4: predictive effects of need satisfaction, difficulties, and service factors	5.33	0.932	0.900	0.067	0.047

Figure 2 demonstrates the results of SEM analyses on the effects of intrapersonal factors on outcome variables (Model 2). Emotional competence had negative predictive effect on negative mental health (*β* = −0.45, *p* < 0.001) and positive predictive effect on well-being (*β* = 0.30, *p* < 0.001). While beliefs of adversity and resilience did not have predictive effect on mental health problems, they positively predicted well-being (*β* = 0.27 and 0.25, respectively, *p* < 0.001). The findings supported Hypothesis 3a and Hypothesis 3b.

The predictive effects of interpersonal factors indexed by family functioning on the outcome variables are shown in Figure 3 (Model 3). Positive family functioning predicted negative mental health negatively (*β* = −0.26, *p* < 0.001), and positive well-being positively (*β* = 0.55, *p* < 0.001). These findings supported Hypotheses 4a and 4b. Meanwhile, the predictive effect of negative functioning of family on negative mental health reached significance (*β* = 0.12, *p* = 0.05), but it did not significantly predict well-being. Only Hypothesis 4c was supported.

The predictive effects of satisfaction of needs, perceived difficulties, knowledge of service, perception of usefulness and evaluation of services on the outcome variables (Model 4) are shown in Figure 4. Need satisfaction negatively predicted negative mental health (*β* = −0.23, *p* < 0.001) and positively predicted well-being (*β* = 0.53, *p* < 0.001). However, knowledge of services, positive perceptions and evaluation of service did not predict mental health problems. In addition, while knowledge of services did not predict well-being, the predictive effects of usefulness of services (*β* = 0.08, *p* = 0.05) and evaluation of services (*β* = 0.07, *p* = 0.062) on well-being were significant/marginally significant. Therefore, Hypotheses 5a and 5b were partially supported. Finally, difficulties positively predicted negative mental health (*β* = 0.49, *p* < 0.001) and negatively predicted well-being (*β* = −0.13, *p* < 0.001). The findings supported Hypotheses 5c and 5d.

To examine gender differences for each model, scalar invariance across gender groups in the measurement model of each SEM model was first examined. The results showed the establishment of the scalar invariance across gender groups for each measurement model (∆CFI < 0.01 when comparing configural model with factor-loading constrained model and when comparing factor-loading constrained model with both factor-loading and intercept constrained model). Second, on the condition of establishment of scalar invariance in the measurement model, multigroup SEM was conducted for each SEM model. Comparison of the configural model with the path coefficients-constrained model showed ∆CFI < 0.01, indicating no gender differences for the four SEM models. The detailed information is shown in Table 6. The predictive effects of predictors in each SEM model in both gender groups were presented in Table 7.

**Table 6:** Model fix indices of the multigroup SEM across genders (including both measurement and structural models).

Model	*χ^2^*/*df*	CFI	TLI	RMSEA	SRMR	∆ *χ^2^*	∆ *df*	∆CFI
Measurement model								
Configural model								
Model 1: effects of COVID stress	3.01	0.914	0.900	0.065	0.055			
Model 2: effects of intrapersonal factors	2.36	0.935	0.922	0.053	0.056			
Model 3: effects of ecological factors	3.47	0.926	0.908	0.071	0.061			
Model 4: effects of need satisfaction, difficulties, and service factors	4.46	0.947	0.915	0.085	0.051			
Factor loadings constrained								
Model 1: effects of COVID stress	2.98	0.912	0.902	0.064	0.057	35.44**	17	0.002
Model 2: effects of intrapersonal factors	2.35	0.932	0.923	0.053	0.058	29.22*	15	0.003
Model 3: effects of ecological factors	3.37	0.925	0.911	0.070	0.062	21.81*	11	0.001
Model 4: effects of need satisfaction, difficulties, and service factors	4.34	0.939	0.918	0.083	0.060	17.88	5	0.008
Intercepts and factor loading constrained								
Model 1: effects of COVID stress	2.95	0.910	0.903	0.063	0.058	35.62**	17	0.002
Model 2: effects of intrapersonal factors	2.32	0.931	0.925	0.052	0.058	21.52	15	0.001
Model 3: effects of ecological factors	3.25	0.924	0.916	0.068	0.063	11.16	11	0.001
Model 4: effects of need satisfaction, difficulties, and service factors	3.89	0.939	0.929	0.077	0.060	1.09	5	0.000
Structural equation model								
Configural model								
Model 1: effects of COVID stress	3.01	0.914	0.900	0.065	0.055			
Model 2: effects of intrapersonal factors	2.36	0.935	0.922	0.053	0.056			
Model 3: effects of ecological factors	3.47	0.926	0.908	0.071	0.061			
Model 4: effects of need satisfaction, difficulties, and service factors	4.04	0.906	0.861	0.083	0.053			
Regression coefficients constrained								
Model 1: effects of COVID stress	3.00	0.913	0.901	0.064	0.057	13.77*	6	0.001
Model 2: effects of intrapersonal factors	2.38	0.933	0.922	0.053	0.061	17.58**	6	0.002
Model 3: effects of ecological factors	3.42	0.926	0.910	0.071	0.062	5.20	4	0.000
Model 4: effects of need satisfaction, difficulties, and service factors	3.73	0.904	0.875	0.077	0.055	14.69	10	0.002

^*^p < 0.05; ^**^p < 0.01.

**Table 7:** SEM model results in different gender groups.

	Male				Female			
	Negative mental Health		Positive well-being		Negative mental Health		Positive well-being	
	*β*	*SE*	*β*	*SE*	*β*	*SE*	*β*	*SE*
Model 1: COVID stress								
Danger and contamination	0.03	0.78	−0.15*	0.07	−0.07	0.79	0.14	0.06
Socio-economic consequences	0.63***	0.89	−0.48***	0.08	0.38***	1.05	−0.30***	0.07
Checking behavior	−0.09	1.06	0.57***	0.11	0.12	1.19	0.17	0.09
Model 2: intrapersonal factors								
Beliefs of adversity	−0.06	0.91	0.20**	0.06	−0.21**	1.17	0.36***	0.06
Resilience	−0.00	1.11	0.27**	0.08	0.15	1.62	0.21*	0.07
Emotional competence	−0.36***	0.99	0.37***	0.06	−0.49***	1.34	0.22**	0.05
Model 3: ecological factors								
Positive family functioning	−0.29***	0.58	0.59***	0.06	−0.27***	0.77	0.47***	0.06
Negative family functioning	0.07	0.78	0.14*	0.07	0.09	1.10	−0.04	0.07
Model 4: need satisfaction, difficulties, and service-related factors								
Need satisfaction	−0.29***	0.64	0.44***	0.08	−0.21***	0.74	0.51***	0.06
Difficulties encountered	0.53***	0.66	−0.16**	0.06	0.46***	0.79	−0.14**	0.05
Knowledge of university services	0.06	0.15	−0.07	0.01	−0.03	0.19	0.08*	0.01
Perceived usefulness of services	−0.08	0.44	0.11	0.05	−0.00	0.58	0.07	0.03
Evaluation of services	0.09	0.62	0.16*	0.06	0.04	0.71	−0.01	0.04

^*^p < 0.05; ^**^p < 0.01; ^***^p < 0.001.

## 4 Discussion

This study investigated the prevalence of mental health illness and positive well-being amongst students in universities in Hong Kong in the pandemic period. The significance of the study is shown in several aspects. First, while the pandemic has significant influence on student mental health, limited research has been done to understand university students’ mental health in Hong Kong under the pandemic. Second, few studies have employed multiple measures of mental health morbidity and positive well-being to examine the related issues in a single study. Third, few studies have been done to explore the role of satisfaction of needs and students’ service utilization and perceptions in college students’ mental health under the pandemic (Shek et al., 2022a,b). Finally, few studies examined the gender differences in the predictive effects of different ecological factors on mental health of university students.

### 4.1 Prevalence of psychological morbidity

The findings of this study indicate the worrying prevalence of mental health symptoms in Hong Kong university students in the pandemic period. Results showed that approximately half of the participants experienced symptoms of anxiety and depression and around one fifth had symptoms of stress, which aligns with previous research (Czeisler, 2020; Shek et al., 2022a). Compared to two studies conducted at earlier stages of the pandemic (i.e., the fourth wave and the fifth wave) (Shek et al., 2022a, 2023b), the prevalence rates of depression and stress are slightly lowered [37.1% (this study) vs. 39.0% (fifth wave) and 40.0% (fourth wave) for moderate-to-above levels of depression and 18.6% (this study) vs. 21.5% (fifth wave) and 22.2% (fourth wave) for moderate-to-above levels of stress]. However, the prevalence rate of anxiety remains relatively stable or was even slightly higher [52.3% (this study) vs. 52.1% (fifth wave) and 50.7% (fourth wave)] for moderate-to-above levels of stress. Compared to the rates in pre-pandemic studies, the levels of mental health symptoms in college students in Hong Kong have significantly increased. Before the epidemic, the rates of anxiety, stress, and depression were, respectively, 41, 27, and 21% (Leung, 2017). However, in this study, the rates were 33.2, 60.8, and 54%, respectively, all of which are higher than pre-pandemic rates. Moreover, these rates surpass those reported in other regions during the pandemic. For instance, a survey of 3,881 college students in mainland China reported an anxiety rate of 26.6% and a depression rate of 21.2% under the pandemic (Chang et al., 2020). Similarly, in Singapore, the rates of anxiety and depression in college students during the pandemic reached 25 and 32%, respectively (Yong and Keh, 2022). In the United States, a study of 2,031 college students under the pandemic found a prevalence of depression and anxiety, respectively, at 48.1 and 38.5% (Wang et al., 2020). Therefore, this study reveals the higher prevalence of mental health symptoms in Hong Kong college students during the pandemic. In accordance with previous empirical literature, the study also identified a prevalence of Internet addiction close to half (47.3%) among Hong Kong college students (Shek et al., 2023a). The findings may possibly be explained by the cumulative stress due to Social Event (Ni et al., 2020) and the very strict anti-pandemic policies in Hong Kong.

### 4.2 COVID-19 stress, psychological morbidity, and positive well-being

Regarding the perceived stress associated with COVID-19, “socio-economic consequences” (SC) predicted mental health symptoms positively and well-being negatively. These findings are in accordance with previous research suggesting the impact of socio-economic consequences on mental health of college students under the pandemic. As many students enrolled in universities in Hong Kong were from the grassroots and some even need to support themselves financially through doing part-time work, the worries about economic and social consequence of the pandemic would become a big stressor to university students, which might trigger their higher mental health symptoms (Busetta et al., 2021; Ghafouri et al., 2022). Besides, under the pandemic, the cost of medicines, masks, and disinfectants, as well as the increased cost of Wi-Fi due to studying online at home, are also stressors for students particularly for those with lower socioeconomic status (Shek, 2020). In addition, students who worry that the pandemic may have a profound impact on the finance of their families may also tend to develop higher mental health problems (Ghafouri et al., 2022). Interestingly, another dimension of COVID-19 stress “danger and contamination” (DC) did not have predictive effect on both well-being and negative mental health, which was contrary to previous research (Czeisler, 2020; Gao et al., 2020). This discrepancy may be due to the stringent health policies implemented in Hong Kong under the COVID-19 pandemic (Chan et al., 2021; Du et al., 2022).

### 4.3 Intrapersonal competencies and mental health

This study found that beliefs of adversity and resilience positively predicted well-being, while emotional competence predicted psychological morbidity negatively and well-being positively. Previous research on Chinese youth has suggested protective role of positive beliefs about adversity in adolescent well-being (Wang and Liu, 2022). This can be explained by the stress mindset theory, which suggests that people’s positive and negative beliefs about stress can affect their experiences and coping strategies in stressful situations: people who hold a positive attitude and view adversity as a personal development opportunity are less likely to experience hardship, while those who hold a negative attitude and believe that stress is harmful are more likely to suffer from this pain (Wang and Liu, 2022). Furthermore, in the context of Chinese culture, individuals with beliefs of adversity are more inclined to attain inner calm during COVID-19 (Shek, 2020). Regarding resilience, previous research has demonstrated that resilience can mediate the association between adverse or stressful events and mental health of Chinese adolescents (Cui and Xie, 2021). A higher level of resilience could make students happier and improve their life on some aspects such as feeling more optimistic, peaceful, clear-minded, and energetic (Huerta et al., 2021). Also, individuals with higher resilience showed higher adaptability and better coping under the pandemic (Demetriou et al., 2020). As for emotional competence, excellent ability to self-regulate thoughts and emotions leads to increased well-being (Huerta et al., 2021). A calmer, more relaxed mind and thoughts can help reduce the stress caused by the pandemic. From a prevention perspective, promoting the positive psychological attributes of university students constitutes a priority in helping university students to thrive under the pandemic.

### 4.4 Family functioning and mental health

Results revealed that positive family functioning was a predictor of both well-being and negative mental health, whereas negative family functioning positively predicted negative mental health but could not significantly predict well-being. In accordance with the previous research (Albanese et al., 2019; Ebrahimi et al., 2019), the current findings suggest the importance of the family system and its support and cohesion for mental health and well-being of university students. Regarding positive family functioning, during the pandemic, it acts as a security blanket for university students to survive and study. In a study on Chinese vocational students, poor family functioning leaded to increased mental health symptoms such as anxiety and depression while good family functioning was associated with improved well-being (Pan et al., 2020). Skeens et al. (2023) found that parental distress predicted worsened quality of life and loneliness of American youth under the pandemic. Combining these literature, findings of this study highlight the importance of promoting the positive family functioning in university students under the pandemic. However, family life education programs are not common in the higher education context.

### 4.5 Perceived need satisfaction, difficulties, and university services and mental health

As expected, need satisfaction was found to predict mental health problems in a negative direction and well-being in a positive direction. On the other hand, perceived difficulties were found to be related to mental health problems positively and well-being negatively. Moreover, perceived usefulness of university services among students were also found to be positively related to well-being. In terms of need satisfaction, previous studies have found a positive relation between need satisfaction and well-being and reported that people who had higher levels of need satisfaction would have enhanced feeling of self-competence, positive mood, vitality (e.g., Šakan et al., 2020), as well as lower stress and decreased negative affect (Yang et al., 2018). This can be explained by the self-determination theory (SDT), which claims that a person’s basic psychological needs need to be satisfied to achieve well-being (Šakan et al., 2020). Our results indicate that with higher satisfaction of needs, university students’ well-being would be promoted. In terms of perceived difficulties, a previous study confirmed that most students (82.2%) believed that the COVID-19 pandemic has brought them some difficulties, and those who perceived more suffering during the pandemic had worse positive moods (Namekata and Yamamoto, 2021). As very few previous studies have shown perceived difficulties have affected students’ mental health under the pandemic (Shek et al., 2022b), this study provides important empirical evidence to enrich the limited literature on the association between perceived difficulties and personal well-being under the pandemic.

Lastly, based on the results of the SEM, students’ services knowledge, service usefulness, and service evaluation, could not predict negative mental health. However, the perceived service usefulness was found to predict the well-being, which suggests that designing effective mental health services is crucial for promoting long-term well-being. The insignificant finding may be due to the fact that first year students were recruited and many of them might not have sought services from the University. Previous findings have indicated that universities can offer services such as counseling and career development services for students to contribute to their well-being (Oldfield et al., 2018). When universities offer effective support and services, they act as protective factors that help students navigate challenges and maintain good mental health. However, to our knowledge, no research has been performed on the effects of university services on students’ mental health and well-being under COVID-19. Therefore, this study provides important empirical evidence which enriches the limited literature.

### 4.6 Gender differences in effects of different predictors

This study showed that there were no gender differences in the effects of different predictors on mental health of university students. This is in line with some existing studies (Hassan et al., 2011; Sergi et al., 2021). One possible explanation is that the gender role has become more egalitarian in the global including many Asian modern countries and districts such as Japan, China, and Hong Kong (Sugihara and Katsurada, 2002; Boehnke, 2011). In addition, as the university of the participants is a public university in Hong Kong which has high requirements in student admission, students from both genders may all possess certain competences and more egalitarian gender perceptions which reduces traditional gender differences. Finally, the insignificance of gender difference might be due to the fact that the data were not collected from random samples.

### 4.7 Implications and limitations

This is a pioneering study which extends our knowledge of first-year university students’ mental health in a Chinese context under the pandemic. The study contributes to the existing limited literature on risk and protective factors of mental health of university students under the pandemic. Theoretically, it contributes to the understanding of the ecological systems theory and positive youth development approach (Bronfenbrenner, 2001; Benson, 2007; Shek, 2007), which highlights the important roles of risk and protective factors in different ecological systems in mental health of university students. Practically, the study highlights the need for addressing negative mental health outcomes in students in higher education in the pandemic period. Also, research findings on the impacts of COVID-related stress, intrapersonal competencies, family environment, satisfaction of needs, and perceptions of university services can provide important information for university policymaking and development of effective prevention and intervention programs for promoting mental health of university students even after the pandemic period. For example, the high prevalence rates of mental health problems and the predictive effects of COVID-19 stress identified in this study highlights the needs for a careful assessment of the COVID-19 dysfunctional cognitive-behavioral coping strategies (Mansueto et al., 2022). The risk of COVID stress and the protective role of different intrapersonal and interpersonal factors also highlights a need to combine or integrate different therapies to reduce mental health problems in university students. These included Cognitive Behavior Therapy or Mindfulness (to reduce negative affectivity and distress) (Cosci et al., 2020) and Well-Being Therapy (to increase well-being, resilience, positive mental health) (Fava et al., 2017; Carrozzino et al., 2022) for improving mental health in university students.

Despite these strengths, there are several limitations of this study. First, as the study was conducted in Hong Kong, China, the findings may not be generalizable to students in other places and cultural contexts. Second, the use of self-reported measures may involve issues of social desirability bias. In addition, our findings did not investigate the long-term effects of negative mental health outcomes on university students. Third, we used composite scores instead of latent variables for predictors in Model 4 due to large item numbers and undesirable model fit for latent variables. Finally, future research should go further to examine complicated relationships between predictors in different models such as potential mediating and moderating roles of intrapersonal factors in relationship between family and other factors and mental health to deepen the understanding.

## 5 Conclusion

This study provides valuable insights into the prevalence and status of mental health and well-being in university students under the COVID-19 pandemic. The findings high-light the risk role of COVID-related stress, negative family functioning, and perceived difficulties and the protective role of psychological strengths (resilience, adversity associated beliefs, and emotional competence), positive functioning of family, and need satisfaction on university students’ mental health. In addition, the importance of positive perceptions and evaluation of university services in university students’ mental health was also highlighted. Additionally, interventions that enhance intrapersonal competencies, positive family functioning, satisfaction of needs, and effective service utilization may protect against negative health outcomes and promote well-being. From a public health perspective, this study is a valuable contribution to the literature on mental health and well-being under the COVID-19 pandemic and provides important insights for future research and practice.

## Data availability statement

The raw data supporting the conclusions of this article will be made available by the authors, without undue reservation.

## Ethics statement

The studies involving humans were approved by the Institutional Review Board (or its Delegate) at the Hong Kong Polytechnic University. The studies were conducted in accordance with the local legislation and institutional requirements. The participants provided their written informed consent to participate in this study.

## Author contributions

DS: Conceptualization, Data curation, Funding acquisition, Methodology, Project administration, Resources, Supervision, Writing – review & editing. WC: Writing – original draft, Writing – review & editing. DD: Formal analysis, Writing – original draft. XL: Writing – original draft, Writing – review & editing. CC: Data curation, Writing – review & editing.
